# Machine Learning Methodology in a System Applying the Adaptive Strategy for Teaching Human Motions

**DOI:** 10.3390/s20010314

**Published:** 2020-01-06

**Authors:** Krzysztof Wójcik, Marcin Piekarczyk

**Affiliations:** 1 Production Engineering Institute, Cracow University of Technology, Al. Jana Pawla II 37, 31-864 Cracow, Poland; 2 Institute of Computer Science, Pedagogical University of Cracow, ul. Podchorazych 2, 30-084 Cracow, Poland; marcin.piekarczyk@up.krakow.pl

**Keywords:** pattern recognition, human–machine interface, machine learning, MEMS sensors, haptic feedback, motor learning

## Abstract

The teaching of motion activities in rehabilitation, sports, and professional work has great social significance. However, the automatic teaching of these activities, particularly those involving fast motions, requires the use of an adaptive system that can adequately react to the changing stages and conditions of the teaching process. This paper describes a prototype of an automatic system that utilizes the online classification of motion signals to select the proper teaching algorithm. The knowledge necessary to perform the classification process is acquired from experts by the use of the machine learning methodology. The system utilizes multidimensional motion signals that are captured using MEMS (Micro-Electro-Mechanical Systems) sensors. Moreover, an array of vibrotactile actuators is used to provide feedback to the learner. The main goal of the presented article is to prove that the effectiveness of the described teaching system is higher than the system that controls the learning process without the use of signal classification. Statistical tests carried out by the use of a prototype system confirmed that thesis. This is the main outcome of the presented study. An important contribution is also a proposal to standardize the system structure. The standardization facilitates the system configuration and implementation of individual, specialized teaching algorithms.

## 1. Introduction

### 1.1. Motor Learning

Motion activity is understood as the performance of a certain movement of parts of the body. Teaching these activities (i.e., motor learning [1]) has great social significance, particularly in areas such as rehabilitation for people with motor system dysfunctions, sports, recreation, as well as the acquisition of skills that are essential for professional vocational work (e.g., piloting, machine operation, and teleoperation) [2,3,4,5,6,7,8,9,10,11]. The possibility of facilitating the teaching of these kinds of activities through automated systems is of enormous significance.

**Figure 1:**
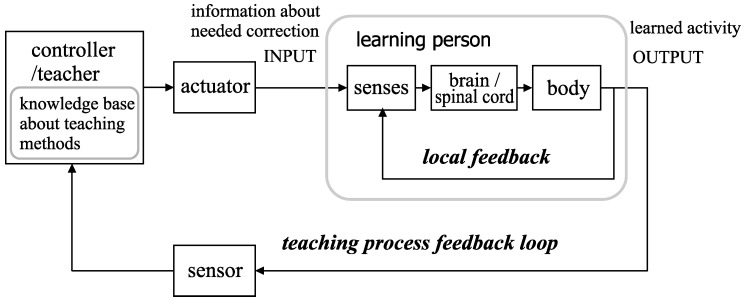
The scheme of the learning process with teacher participation. The motion activity of the learner’s body is evaluated by the teacher or automatic controller, which uses an actuator to send feedback to the learner. The object of this process (i.e., the learner) uses the local feedback loop to control his or her movements.

First, we examine the general scheme of learning. In the majority of known strategies, this process takes place with the participation of a teacher (supervised learning), usually with the use of a feedback loop. The process is illustrated by means of a simplified chart in Figure 1.

With the use of local feedback, the learner controls the activity that is the subject of learning (e.g., moving specific parts of the body or voice emission). Moreover, from the point of view of control theory, the learner (as a whole) is also an object of control with a designated input and output [12]. The teacher’s suggestions are the input signal, while the output signal is the activity undertaken by the learner (e.g., a movement or sound). This activity is assessed by the teacher, who determines the best means for its correction and subsequently sends the information to the learner. Thus, we see another, more general feedback loop connected to the process of learning. The teaching process must have a defined general aim that should refer to further learning outcomes, such as the improvement of a person’s health or an increase in the efficiency of machine operation. The appropriate assessment of these outcomes requires the learning process to be conducted using expert knowledge (we consider an expert to be a person who is able to establish the proper way of teaching and understand technical aspects of the system).

The introduced general scheme can be utilized to describe the automatic system for learning fast motor skills. The system should be characterized by certain features and satisfy specific requirements, namely:

1. The motion sensor system should be convenient and non-obstructive to the learner [3,13].
2. The learning method should feature only short delays between the performance of the movement, the trainer’s communication, and the reaction of the learner (Concurrent Feedback (CFB)) [10,13].
3. Teacher–student communication should be performed at a relatively low level, which does not require a great deal of intellectual activity on the part of the learner [4,14].
4. Teaching methods should be adaptive, i.e., they should be able to adapt automatically to the specific teaching conditions and changing phases of the entire teaching process [13].

The above requirements define the main problems related to building an automatic system for teaching fast and periodic movements. Such problem identification has not been described in the literature. Nevertheless, there are works in which similar problems are discussed [5,6,15,16,17,18,19,20,21,22,23,24,25]. We return to this matter in Section 1.5 (Related Work).

### 1.2. The Motion Capture Process

Systems for motion capture can utilize many kinds of sensors. For instance, by using image sensors (cameras) and algorithms for image recognition, we are able to determine a person’s profile in the image and calculate the coordinates of selected parts of the body [26]. MEMS (Micro-Electro-Mechanical Systems) sensors are frequently used as motion sensors [17,27]. The small casing of a typical MEMS unit can contain several kinds of sensors, such as triaxial accelerometers, gyroscopes, and magnetometers. These sensors make it possible to calculate the acceleration of body parts with respect to a certain coordinate system (e.g., associated with the Earth). As a result, the speed and position can be computed.

### 1.3. The Communication between Teacher and Learner

Teacher–learner communication can be achieved through different senses, such as hearing, sight, touch, proprioception, and balance. Given the above mentioned characteristics of motor learning, most currently existing systems utilize a combination of touch, auditory, and visual sensations [14]. Especially in systems that apply concurrent feedback, because of the short reaction time, the sense of touch is preferred (systems with Vibrotactile Feedback (VTFB) [4,13,18]). The literature describes many useful constructions that generate haptic and vibrotactile sensations (e.g., vibration motors). In accordance with control theory, herein, these devices are called actuators [12].

### 1.4. Classification Processes in Teaching Algorithms

In the decades of practice in motion teaching, many diverse strategies have been invented [1,2]. However, as previously noted, the adequate choice of strategy requires knowledge and experience. The acquisition of expert knowledge is a central problem that faces the creators of automated motor learning systems. It is characteristic that capable systems are involved in the domain of rehabilitation, not in sports and professional skills, in which proper teaching techniques need to be respective and flexible [3,13]. There exist many factors (e.g., learner’s fatigue level and injury) that affect the undertaken movement to a significant degree. Thus, in order to select an appropriate teaching algorithm, a classification of the motion signals has to be conducted. The knowledge that allows this process to be performed can originate from an expert or an experienced teacher. The expert can examine a group of sample signals and ascribe them to previously defined classes. Consequently, a sequence composed of pairs (signal, label_of_class), called a learning sequence, is built [28].

The automatic assignment of an unknown signal (signals are also called objects) to individual classes can be assessed using a predefined metric function. This technique is utilized in the Nearest Neighbor (NN) method and its modifications (e.g., k-Nearest Neighbor (kNN); see Section 2.3) [28,29,30,31]. Motion signals can also be classified using methods in which the probability that an object belongs to a particular class is estimated (e.g., Bayesian classifier [27,28]). The Hidden Markov Model (HMM) is often utilized for this purpose [32]. The signals can also be recognized using a model based on a defined ontology [33]. A syntactic method can be used as another approach. If an object can be described with the aid of particular grammar, then we can consider the object to belong to the class ascribed to this grammar [34]. Other commonly used classification methods for motion signals apply Artificial Neural Networks (ANNs) [17,28,35]. Similarly, methods based on Support Vector Machine (SVM) are frequently used [21,36].

The above points show a wide range of available solutions. However, we should be aware that the method applied in motor learning systems must be understandable to the teacher and possible to implement in real-time control algorithms.

### 1.5. Related Work

There are many works that describe particular subproblems concerning the motion activity teaching. They mainly involve the implementation of learning systems in rehabilitation (in which movements are generally slow) and sports [3,18]. The main properties of typical teaching systems are presented in Table 1. The depicted solutions do not meet all the requirements listed in Section 1.1. The systems proposed by Stamm [6] and Wang et al. [22] are typical examples. The systems, using a set of sensors, calculate several parameters that describe the motion. However, the data interpretation is performed by the trainer, and the feedback is provided to the learning person after the motion.

The description of the learning person in terms of an object being controlled (see the flowchart in Figure 1) suggests that for the motor learning, some methods normally applied to machine learning problems can be utilized. This particularly relates to teaching the ANN and embedded agents [37]. The problems of applying diverse teaching strategies and ways of teacher–learner communication are broadly described in the literature. Despite the fact that the works do not involve the real-time motor learning, many concepts (e.g., teachable agent [38]) may be adopted in this field [39,40].

### 1.6. Study Objectives

The described properties of an automatic teaching system suggest that the system efficiency depends on several cooperating subsystems. Therefore, the question is not what kind of sensors should be used, nor what particular algorithms for signal analysis have to be implemented. Instead, the following main research question is posed:

Can fast motion activities be efficiently taught using an automatic motor learning system that:

- uses an array of MEMS inertial sensors,
- utilizes vibrotactile actuators to ensure system–learner communication, and
- applies the classification process to select the adequate teaching algorithm?

The goal of the presented paper is to provide an answer to this question.

In an effort to achieve this aim, a prototype motor learning system was designed and built. The constructed system can be treated as a platform for testing numerous variants of system elements. During preliminary tests, several algorithms for teaching were examined, and a typical motion activity was chosen to be taught. Finally, the interaction between the teaching system and the learners was examined. Two groups of subjects were taught using the automatic system, and the learning process was evaluated using defined efficiency factors. A statistical analysis of the obtained data was performed. To enable similar systems to be built and the whole experiment to be replicated, we describe key elements of the system implementation. This description is an additional contribution of this paper. However, because of space limitations, we do not describe matters that are beyond the main scope of our study.

In Section 2, we describe the main problems related to the classification task. Section 3 presents the results of implementing the system. In Section 4, we summarize the main outcome of this research.

## 2. The Motion Activity Teaching System

**Figure 2:**
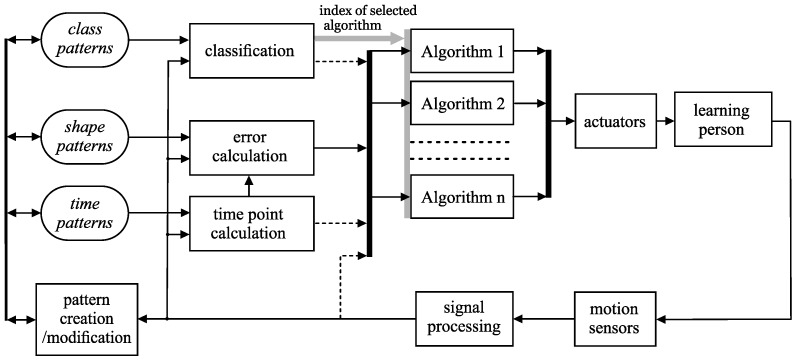
General flowchart of the real-time teaching system prototype. The signal that selects the teaching algorithm is symbolized by the thick gray arrow.

Figure 2 presents a simplified diagram of the signal flow in the prototype teaching system. The main differences from the general scheme in Figure 1 are the application of a set of teaching algorithms instead of a single algorithm and the implementation of modules intended for signal classification. These elements are described in the next sections.

The proposed learning system is a real-time discrete control system (the sampling interval equals 10 ms). In each period, a sequence of actions is performed: reading the signals from sensors, signal preprocessing, classification, and realization of the particular algorithm with relevant control of the actuators.

### 2.1. The Motion Sensors and Preprocessing of Their Signals

With the aim of building a portable system, we resolved to use MEMS inertial units (their particular type is described in Section 3). We assumed that the sensors, which were attached to selected places on a learner’s body, sent digital signals of acceleration and rotation directly to the main minicomputer. The transmitted values were represented by floating point numbers. Thus, the components of acceleration formed a vector, $a={\left[a_{x}\;a_{y}\;a_{z}\right]}^{T}$, which, after applying integration operations, could be utilized to calculate speed and position signals. The rotation was sent by means of the four values creating the quaternion $q=(v_{x},v_{y},v_{z},k)$ [31,41]. This represents the rotation of the sensor in relation to its fixed, initial orientation. Consequently, we assumed that the quaternion expressed the sensor rotation in relation to the coordinate system associated with the Earth. However, the acceleration vector was represented in the local sensor frame. As a result, even a small, accidental rotation of a part of the body during the course of the movement significantly changed the values of the measured accelerations. This could be remedied by employing the value of the rotation supplied by the quaternion and calculating the acceleration in the independent, connected with the Earth, frame. We call this frame simply the inertial frame.

Now, let us describe the motion more precisely. From the quaternion q, we can calculate a matrix A that describes the sensor’s rotation. On this basis, we can designate a matrix B that defines the conversion of coordinates from the sensor frame to the inertial reference frame: $B=A^{-1}$. Then, we can obtain the sensor acceleration expressed in the inertial frame: $a_{iner}=B\;a$. Finally, to determine the motion from the obtained vector of acceleration, we must deduct the constant value of gravitational acceleration from it. Let $a_{motion}$ denote the acceleration vector after this modification.

We must be aware of important problems involving the accuracy of the computed motion signals. These problems are the result of accelerometer errors, but are primarily associated with the phenomenon of the gyroscope drift [41,42]. Due to space limitations, we do not discuss these issues.

Let us consider one chosen component of the vector $a_{motion}$ received from a given sensor. We regard this component as a certain signal, and we ascribe to it a unique index *i*.

The values of this signal, sampled at discrete time stamps, create a time series that is denoted by $F_{i}$; that is,

(1) $$F_{i}=\left(f_{i}^{1},f_{i}^{2},\ldots ,f_{i}^{m}\right);\;i\in I$$

where $f_{i}^{k}$ is the value of the $k^{\mathrm{th}}$ probe of the $F_{i}$ signal (we adopted the rule that the upper index relates to the probe number), m is the number of signal probes, and I is the set of indices that identify the signals.

Signals in the form of Expression (1) are referred to as one-dimensional signals. Typically, we have several one-dimensional signals at our disposal. The selection of the most appropriate signals for further analysis is performed by the expert using a dedicated program interface. The set of indices of selected signals is denoted by K, i.e., K⊂I.

All selected one-dimensional signals should be filtered to reduce noise and disturbances. We can assume that the noise density of the acceleration signal has an approximately constant value (for the MEMS accelerometer in the prototype system, this value was 0.4 mg/$\sqrt{\mathrm{Hz}}$ [41]). Thus, for our application, which involves relatively slow signals, we can significantly reduce noise using a low pass filter (for a cutoff frequency of 5 Hz, the noise amplitude was reduced by approximately 10 dB). In the described system, a simple low pass IIR (Infinite Impulse Response) filter [43,44] was applied: $o^{k}=\left(1-\alpha \right)o^{k-1}+\alpha f^{k}$, where $f^{k}$ and $o^{k}$ are the values of the $k^{\mathrm{th}}$ probes of input and output signals, respectively, α is the fixed parameter that determines the cutoff frequency.

### 2.2. Choosing the Classification Method

The problem of selecting a proper classification method for motion analysis has been extensively discussed in the literature [21,32,36,45]. The recommended solutions are based on SVM, kNN, HMM, and, in some cases, ANN approaches. Since it is assumed that the expert should actively participate in the process of knowledge acquisition in the motor learning system, the representation of system data should be as simple as possible. For example, the information contained in the parameters of SVM or in the synapse weights of ANN is difficult to interpret. In contrast, an emission matrix used in the HMM method and pattern signals used in the minimal distance methods are understandable to people. Preliminary testing was used to examine the possibility of applying HMM and minimal distance approaches. The motion signals and the general test layout were as described in Section 3. Table 2 presents typical results that were obtained after the analysis of a nine minute motion signal (classification errors were calculated by comparing the outputs of the classifiers with the expert’s judgments; the implementation of HMM was based on [32] and [46]). The error rate of the compared methods was approximately at the same acceptable level, whereas the HMM method (more precisely, a forward procedure [46]) was characterized by a radically shorter computational time. However, for the sake of better supervising the system at work, which was essential for prototype development, we decided to implement a version of minimal distance kNNModelmethod [30] (the method is described in the next section). We return to the potential use of the HMM method in Section 3.7).

### 2.3. Signal Patterns and Definition of the Distance Function

As a result of signal preprocessing, we obtained a set of one-dimensional signals that corresponded to individual sensors and particular components of acceleration, speed, or position. This set is designated S; that is,

(2) $$S=\{S_{i}:\;i\in K\}$$

where $S_{i}=\left(s_{i}^{1},s_{i}^{2},\ldots ,s_{i}^{m}\right)$ is a preprocessed one-dimensional signal, $s_{i}^{k}$ is the value of the $k^{\mathrm{th}}$ probe of the $S_{i}$ signal, and m is the number of signal probes.

The S set will be called a multi-dimensional signal. We defined a one-dimensional pattern as a generalized one-dimensional signal, i.e., a series of probes, and we assumed that they covered only one period:

(3) $$P_{i}=\left(p_{i}^{1},p_{i}^{2},\ldots ,p_{i}^{w}\right);\;i\in K$$

where $P_{i}$ is a one-dimensional pattern, $p_{i}^{k}$ is the value of the $k^{\mathrm{th}}$ probe in $P_{i}$ pattern, and w is the number of pattern probes (equal to the pattern period).

The pattern $P_{i}$ is constructed on the basis of the signal $S_{i}$. A set of one-dimensional patterns, denoted by P, is called a multi-dimensional pattern (by analogy with the one- and multi-dimensional signal). The method of pattern creation is described in Section 2.5.

We now concentrate on the learning sequence, which is the series of exemplary objects and the class labels ascribed to them. If the objects are considered to be multi-dimensional signals of motion, then the learning sequence can be expressed as follows:

(4) $$L=(\left(S_{1},\iota_{1}\right),\left(S_{2},\iota_{2}\right),\cdots ,\left(S_{q},\iota_{q}\right))$$

where $\left(S_{u},\iota_{u}\right)$ is the $u^{\mathrm{th}}$ element of the learning sequence, $S_{u}$ is a multi-dimensional signal, $\iota_{u}$ is a class label, and q is the number of pairs in the learning sequence.

By replacing all $S_{u}$ multi-dimensional signals in L with the patterns created by them, we obtain a sequence:

(5) $$P=(\left(P_{1},\iota_{1}\right),\left(P_{2},\iota_{2}\right),\cdots ,\left(P_{q},\iota_{q}\right))$$

where $P_{u}$ is a multi-dimensional pattern created from the multi-dimensional signal $S_{u}$. The other denotations are as described in (4).

The P sequence represents more general and less redundant knowledge about the teaching motion. Let us consider the $u^{\mathrm{th}}$ multi-dimensional pattern $P_{u}$ from P. This pattern can contain several one-dimensional patterns that relate to individual signals. For the sake of clarity, we introduce the double indexing of one-dimensional patterns. Thus, the one-dimensional pattern $P_{i,u}$ is constructed on the basis of the $i^{\mathrm{th}}$ signal (i∈K) and corresponds to the $u^{\mathrm{th}}$ element of the sequence P. Accordingly, the multi-dimensional pattern $P_{u}$, referring to the $u^{\mathrm{th}}$ element of P, can be expressed as:

(6) $$P_{u}=\left\{P_{i,u}:\;i\in K\right\}$$

Our task is to classify a multi-dimensional signal that describes the present motion. We denote this current signal by $S_{cur}$, i.e.,

(7) $$S_{cur}=\left\{S_{i}:\;i\in K\right\}$$

where $S_{i}=\left(s_{i}^{1},s_{i}^{2},\ldots ,s_{i}^{m}\right)$ is a one-dimensional signal (denotations are as in Equation (2)).

We assumed that in each one-dimensional signal $S_{i}$, signal probes from a certain moment to the present moment of time were memorized (the last probe was indexed by *m*).

Let us turn to the main process of the classification. In order to classify an unknown object (i.e., the multi-dimensional signal $S_{cur}$), we computed its distance, defined by a certain function dist (see (9)), to all patterns in the sequence P. Let us take into account a set of *k* elements from the P, which include the nearest patterns. This set is divided into subsets with the same class label. Each subset can be expressed by:

(8) $$Q_{r}=\left\{\left(P_{z},\iota_{z}\right):\;\iota_{z}=r\right\}$$

where *r* is the class label of all elements included in $Q_{r}$.

The output class is established on the basis of such a subset $Q_{max}$ that has the greatest cardinality.

**Figure 3:**
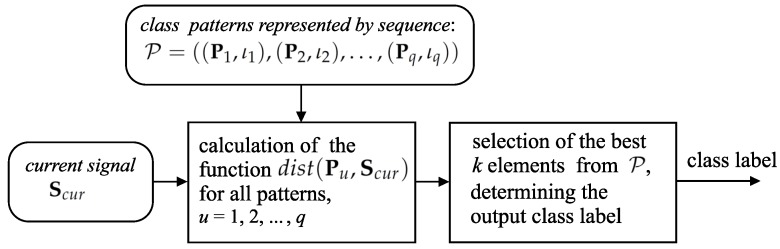
Simplified flowchart of the kNNModel method and data structures utilized in the implementation of this method.

The described algorithm refers to the kNNModel method [30]. Figure 3 depicts the main data structures that are applied in the presented method’s implementation.

Naturally, the properties of the method depend on the definition of the pattern objects and the distance function. In a situation in which the function’s arguments are a multi-dimensional signal and pattern, that is finite sets, the distance (metric) can be defined by employing a certain distance function *h* between the elements of these sets, i.e., between a one-dimensional signal and one-dimensional pattern. With this approach, the problem of computing the dist function can be divided into easier subproblems in which the distances between one-dimensional signals are computed. In the prototype system, the dist function is defined using a simple arithmetic mean:

(9) $$dist\left(P_{u},S_{cur}\right)=1/N\sum_{i\in K}h(P_{i,u},S_{i})$$

where $P_{u},S_{cur}$ are the multi-dimensional pattern and signal, N is the cardinality of set K, and $h(P_{i,u},S_{i})$ is the distance function between the one-dimensional pattern $P_{i,u}\in P_{u}$ and the one-dimensional signal $S_{i}\in S_{cur}$. 

The employed distance function *h* should assess a certain similarity of the sequence of signal samples to those of the pattern. For this purpose, the Dynamic Time Warping (DTW) method [47] was frequently used. However, it applies nonlinear signal scaling, which can result in a discrepancy in the assessment of the signal similarity conducted by the teacher and the system. This makes subsequent pattern editing (Section 2.5) more difficult. In the prototype system, we applied a method based on direct comparison of signals, with the assumption that the degree of similarity ought not to be affected by the linear transformations of the signals (property of translation and scale invariance). To this end, we introduced an auxiliary function *g* that evaluates the distance between the signals after certain scaling and dislocation of one of them. We used the mean squared measure, which is commonly used to compare temporal sequences [43]. The function *g* is defined as:

(10) $$g\left(P_{i,u},S_{i},a,b,c,d\right)=1/n\sqrt{\sum_{k=0}^{n-1}{\left(p_{i}^{b-ak}c+d-s_{i}^{m-k}\right)}^{2}}$$

where $P_{i,u}=\left(p_{i}^{1},p_{i}^{2},\ldots ,p_{i}^{w}\right)$ is a one-dimensional pattern, $S_{i}=\left(s_{i}^{1},s_{i}^{2},\ldots ,s_{i}^{m}\right)$ is a one-dimensional signal, n is the number of probes for which the signals are matched, n≤m,n≤w, and a,b,c,d are the chosen values of parameters; we assumed that they belonged to established sets, namely $a\in Z_{a}$, $b\in Z_{b}$, $c\in Z_{c}$, and $d\in Z_{d}$; see Section 2.4 (for greater clarity of the notation, the operation of transforming the value of the expression b−ak to the closest integer is omitted; similarly, the appropriate shift of this value by the period value (so that it belongs to the range 〈1,w〉, in which the pattern sequence is defined) is also omitted).

The parameters *a* and *b* correspond to the scaling and shift of the signal in the time domain, whereas *c* and *d* are associated with the scaling and shift of the signal values. By using the function *g*, we can define the final metric *h*, which gives the distance between the current one-dimensional signal and the one-dimensional pattern considering their best matching:

(11) $$h\left(P_{i,u},S_{i}\right)=min_{a\in Z_{a},b\in Z_{b},c\in Z_{c},d\in Z_{d}}\;g\left(P_{i,u},S_{i},a,b,c,d\right)$$

The calculation of this function requires the designation of the values of four parameters (a,b,c,d) that minimize the function *g*. Apart from the particular form of the *h* function, the presented method illustrates the general idea of signal and pattern matching and obtaining the fit parameters.

### 2.4. The Calculation of the *h* Function

Let us comment on the numerical aspects of the *h* function computation. This calculation refers to minimization of the *g* function. In the prototype system, a stable two stage “trial and error” algorithm was used [48]. In the first stage, the method consists of computing the values of *g* function for a certain number of possible values of the parameters a,b,c, and *d*. We assumed that the parameters were chosen from the discrete subsets: $Z_{a}^{\prime },Z_{b}^{\prime },Z_{c}^{\prime },Z_{d}^{\prime }$, of the sets $Z_{a},Z_{b},Z_{c},Z_{d}$, respectively (11). Then, the maximum number of necessary computations of the *g* function is the product of the cardinality of these subsets. In practice, we obtained a number on the order of a quarter of a million (in our case, this was achieved for the following cardinalities: card($Z_{a}^{\prime }$) = 30, card($Z_{b}^{\prime }$) = 20, card($Z_{c}^{\prime }$) = 15, and card($Z_{d}^{\prime }$) = 25). The number of the relevant values of parameters *c* and *d* can be reduced by the proper normalization of the signals. The normalization involves the linear transformation of the signal: $s_{norm}^{k}=\zeta s_{pre}^{k}+\eta$ where $s_{pre}^{k}$ and $s_{norm}^{k}$ are the values of the $k^{\mathrm{th}}$ signal probes before and after normalization, respectively, and ζ, η are constant parameters. The normalization was performed in the range in which the signals were compared; see (10). The parameters ζ and η were selected in such a way that the minimal and maximal values of the normalized signal related to established levels (−0.6 and 0.6 in our case). As a result of the normalization process, the number of computations of *g* function could be reduced to an acceptable level (around 5000). The described stage of calculation allowed determining approximate areas that included local minima of the function *g*.

The second stage of the “trial and error” algorithm involved a repetition of the finding procedure in the mentioned areas. This made it possible to locate the minimum more precisely.

The uncomplicated form of the *g* function (it depended on simple $p_{i}^{b-ak}c+d-s_{i}^{m-k}$ components (10)) enabled using an effective program code written in the time optimized assembler language. Consequently, the computation time of *g* function was limited to satisfactory level on the order of 3 ms (for the processor used; see Section 3.2).

### 2.5. The Creation and Updating of Patterns

In this section, we concentrate on the problem of creating class patterns. Under the expert’s supervision, the learner performs a sequence of a dozen to several dozen movements belonging to a particular class. All signals are recorded in the system memory. Let us consider a certain one-dimensional signal. First, this signal is segmented into windows that correspond to the periods of motion [49]. Then, an averaged signal shape is created from these windows (a simple arithmetic mean is employed). Subsequently, windows with the greatest distance (according to the *h* function) from the averaged signal are rejected. Averaging of the remaining signals creates a signal pattern.

The described method corresponds to a simplified *k*-means algorithm of clustering (only one cluster is created) with the use of *h* as a metric function [29,50]. The method was performed for each one-dimensional signal; as a consequence, a multi-dimensional pattern could be be built.

**Figure 4:**
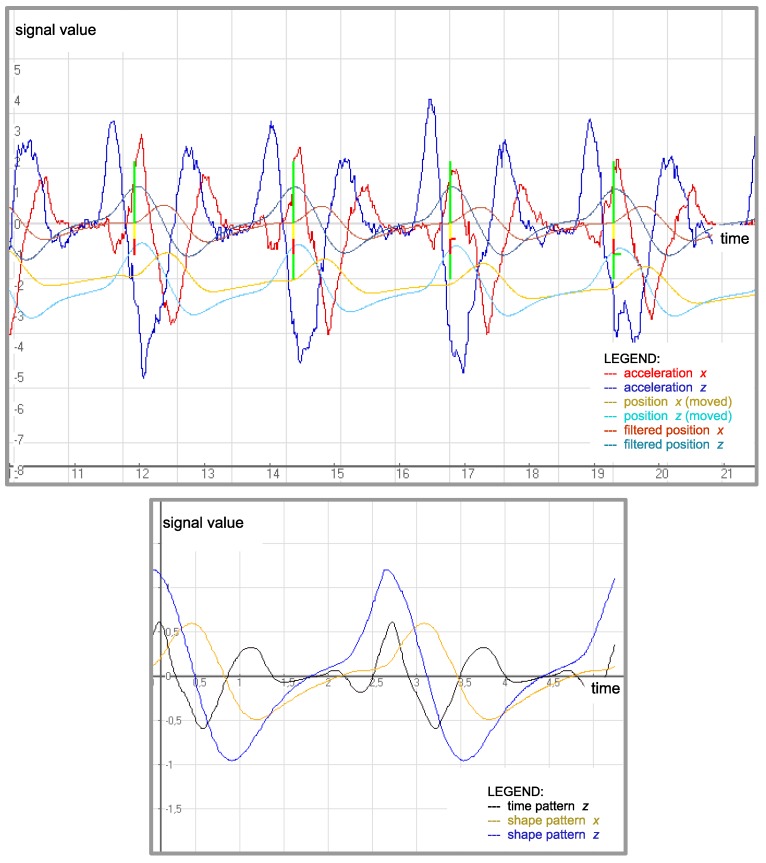
Signals and patterns created from the signals. For example, the red signal in the upper window represents the *x* component of acceleration. The unit of the vertical axis is 1 m/s${}^{2}$, and the time axis is scaled in seconds. The bottom window depicts the time pattern (black) created from the *x* component of acceleration on the base of several periods depicted in the top. Two shape patterns created from the *x* and *z* components of position are also depicted.

Through the creation of new patterns, the expert was able to modify the system knowledge, adapting it to subsequent stages of learning. Additionally, an uncomplicated form of the patterns (simple time sequences) enabled their adjustment to the learner’s individual features. For example, we could adapt the system to the length of the learner’s limbs by changing the amplitude of the pattern signal. Figure 4 illustrates examples of motion signals and the patterns that they create.

### 2.6. The Synchronization of Patterns

In the majority of cases, the teaching algorithms calculate the value of motion error. Thus, besides knowing the signal value at the current moment, we have to know the value of a certain pattern signal that describes the motion. We call this kind of pattern a shape pattern. However, to synchronize the shape pattern properly with the current signal, we should know which probe in the shape pattern corresponds to the current moment. The index of this probe will be called a “time point”. To calculate its value, we can utilize information provided by available motion signals. The time point can be obtained through the appropriate matching of the current signals to special patterns by minimizing the *g* function. This minimization leads to the determination of the parameters a,b,c, and *d* (10). The parameter *b* refers to a shift in the time domain. Moreover, as seen in the (10), this parameter directly corresponds to the last sample in the current signal (see the sample indices for *k* = 0). Thus, the value of the parameter *b* relates to the time point.

**Figure 5:**
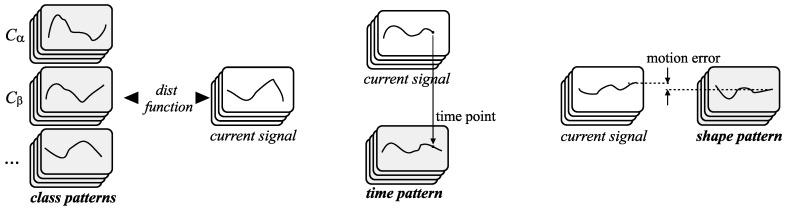
The multi-dimensional signals and patterns applied in the motion learning system: the current multi-dimensional signal and the collection of multi-dimensional class patterns for the classification process (left side), current multi-dimensional signal and multi-dimensional time pattern, and current multi-dimensional signal and multi-dimensional shape pattern.

The special pattern used in the synchronization process is called a time pattern. Similarly, the pattern used in the classification task is called a class pattern. Figure 5 illustrates the introduced types of patterns.

The time pattern can be (but does not have to be) the shape pattern or the class pattern simultaneously. An appropriate decision in this regard was made by the expert.

The multi-dimensional time pattern, which is composed of several one-dimensional time patterns, can be used to determine one reliable value of the time point. We can rank the results of individual solutions that are calculated on the base on one-dimensional time patterns according to a certain reliability criterion. In the described prototype system, the criterion is based on the value of the distance function *h* (11) between the current one-dimensional signal and its associated time pattern. The value of the aggregate time point is calculated as a weighted average of a fixed number of the best results.

### 2.7. The Calculation of Motion Error

Here, we reflect on the problem of calculating the error signal. In the simplest case, the error can be calculated as the difference between the current motion signal value and the value of the sample of the relevant shape pattern, which corresponds to a given moment in time. However, we can significantly reduce the susceptibility to disruption by calculating a weighted average of the errors computed in the “last” samples (e.g., for *n* samples, the weight parameters can vary from zero for the first sample, to one for the last sample taken). Using this method, we can calculate the errors for all of the one-dimensional signals for which the patterns of shape were created. Consequently, we obtain a vector of errors: $e={\left[e_{1}\;e_{2}\;\ldots \;e_{n}\right]}^{T}$. The tasks of calculating the errors and the time points are depicted in the block diagram in Figure 2.

### 2.8. Actuators and Calculation of Their Activity

**Figure 6:**
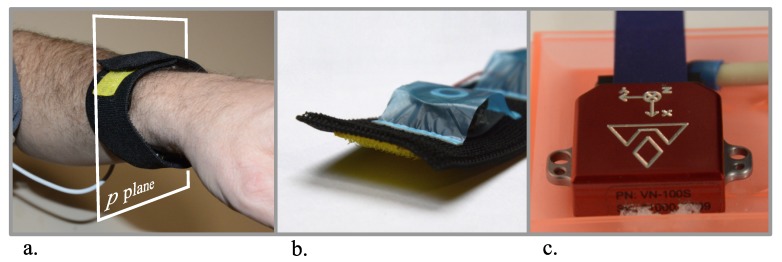
Peripheral elements of the teaching system. (**a**) Actuator: the band built on the base of an elastic hook-and-loop strip. (**b**) Actuator’s units (behind a protective film). (**c**) VN-100 inertial sensor.

Because of the small dimensions and the ability to convey messages quickly, the proposed system used vibrotactile actuators [18]. Before further discussion, we provide some details about the actuator’s construction. Each actuator consisted of four units, which were built using electrodynamic devices (a movable coil and permanent magnet). The vibrations generated by the unit’s coil were passed to the skin surface by the use of a special plastic tappet. The units were mounted onto elastic tape, which constituted a kind of band. Figure 6a,b shows a general view of the actuators.

The actuators were controlled by means of a specialized driver (the prototype system used the Atmega 128 microcontroller). Its task was to convey power impulses to the entry of the relevant unit of the given actuator, according to commands received from the main minicomputer via the USB port (the driver can control two actuators). Normally, a power signal consists of two short pulses with a duration of 0.25 s and a frequency of 20 Hz.

The vibrotactile units were installed on one plane, denoted by *p*, which was perpendicular to an assumed axis of a given body part (see Figure 6a). Turning on the vibrations in one unit informed the learner about the required direction of motion [18].

Let us focus on the means of controlling the actuators. We should define which one of the calculated components of the error vector e (Section 2.7) influences the relevant actuator. An additional issue is determining the means of aggregating many signals into a single actuator signal; the method should be easily understandable to the expert. Therefore, the signal of each $i^{\mathrm{th}}$ actuator is simply a linear combination of the components of the error vector; that is,

(12) $$g_{i}=C\;e=\left[\begin{array}{ccc} c_{11} & \ldots & c_{1n}\\ c_{21} & \ldots & c_{2n}\\ c_{31} & \ldots & c_{3n} \end{array}\right]e$$

where $g_{i}={\left[g_{ix}\;g_{iy}\;g_{iz}\right]}^{T}$ is the vector of the $i^{\mathrm{th}}$ actuator (in the inertial reference frame), $e={\left[e_{1}\;e_{2}\;\ldots \;e_{n}\right]}^{T}$ is the vector of error, n is the number of components of the error vector (equal to the number of shape patterns), and C is a 3×n matrix of weight coefficients of linear combinations.

The main advantage of the above approach is the ability to customize the configuration of the sensors and actuators. The vector e expresses the error in the inertial frame; therefore, the calculated vector $g_{i}$ is also expressed in this frame. However, in order to control executive units, $g_{i}$ must be transformed to a local actuator’s frame. Typically, the actuators are mounted close to certain sensors (they are “rigidly” connected to them). Then, using the rotation matrix of the sensor, we can express the vector $g_{i}$ in the actuator’s frame:

(13) $$w_{i}=A\;g_{i}$$

where A is the matrix of sensor rotation calculated at earlier stages of processing (see Section 2.1), and $w_{i}$ is the vector activating the $i^{\mathrm{th}}$ actuator expressed in its local coordinate system.

Finally, we can project the vector $w_{i}$ onto the plane *p* of the actuator and obtain the two-dimensional vector $o_{i}$, which defines the way of activating its units. We describe the projection operation as follows:

(14) $$o_{i}=G\;w_{i}$$

where $o_{i}$ is the two-dimensional actuator vector on plane *p*, $w_{i}$ is the three-dimensional actuator vector, and G is the 2×3 linear transformation matrix.

In practice, as a result of certain limitations in the way in which the actuator is mounted, there may occur a rotation of its axes in relation to those of the sensor. This effect can be easily compensated by selecting elements of the matrix G in such a way that the simple projection is expanded into a rotation operation.

The next procedure is evident: the only remaining step is to calculate the direction of the vector $o_{i}$ (on the plane *p*) that determines the vibrotactile unit to activate. Unfortunately, before this final step, which conventionally “closes” the feedback loop, we have to resolve several significant problems connected to the physiology of senses and the psychology of learning. We present the most important among them [1,51].

1. A person’s inability to properly interpret the fast changing signals of the actuator; this obvious and fundamental constraint is especially apparent in the detection and interpretation of the actuator signals, which indicate, in succession, different directions of movement.
2. The need to select the optimal parameters of vibrotactile unit impulses so the learner can best perceive and interpret them [18].
3. The requirement to limit the number of actuators activated in a single period of motion; usually, during the course of a single motion period, the learner is able to interpret the signals derived from only a single actuator correctly (the remaining actuators exert a disturbing effect).

### 2.9. Algorithms of Motion Teaching Corresponding to Particular Classes

The calculated vector e can be regarded as a conventional error signal. However, we have at our disposal many kinds of signals and values characterizing the motion (rotation matrices, time point value, *h* distance function values, etc.) that can be used in several specialized teaching algorithms (this possibility is depicted in Figure 2 by dashed arrows). To reveal some essential questions, we focus here on only two algorithms, which correspond to two classes of motion signals. The first class, denoted by $C_{\alpha }$ (see Figure 5), refers to movements in which there are small errors in the taught trajectories. The second class, named $C_{\beta }$, refers to certain typical and often significant errors in the motion.

#### 2.9.1. Algorithm of Class $C_{\alpha }$

The algorithm of the $C_{\alpha }$ class corresponds to the work of a discrete regulator. In each sampling interval, the vectors $o_{i}$ (14) for all actuators are derived. Then, the actuator that should be activated is determined. The selection criterion is based on the maximum length of the actuator vector over one period (the vector length refers to the Euclidean norm). Let $o_{act}$ denote the vector of the activated actuator. Next, the actuator’s unit, which relates to the direction of the vector $o_{act}$ on the plane *p*, is chosen. If the length of the $o_{act}$ exceeds a defined threshold, denoted by len, and the time elapsed since the previous actuator activation overruns a fixed level elaps, then the unit is activated (in the prototype system, the above parameters were experimentally determined: len = 0.03 m, elaps = 2.5 s). After establishing the indices of activated actuator and its unit, the teaching algorithm sends these data to the actuator driver. It generates power impulses that are conveyed to the relevant unit; see Section 2.8.

**Figure 7:**
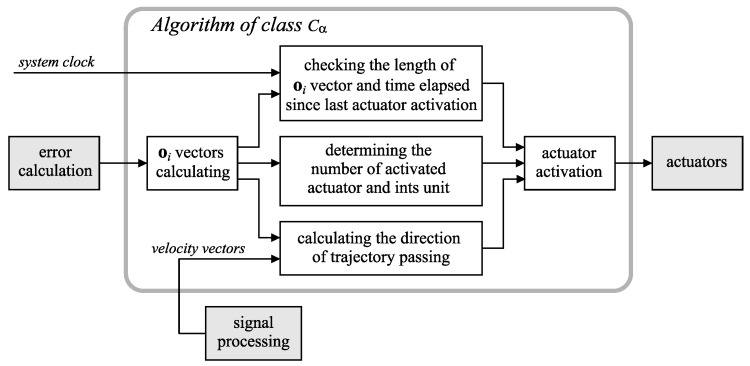
A simplified diagram of the signal flow of the class $C_{\alpha }$ algorithm. The elements of the general system (Figure 2) are depicted in gray.

In our case of teaching fast motions, the key problem was the inability of the learner to interpret rapidly alternating signals (see the constraints described in Section 2.8). However, with the assumption that the taught trajectory is a closed curve, the generation of actuator signals may be limited only to cases in which the considered body part exceeds the trajectory from the inside. The direction of a velocity vector is used to detect such situations. The learner should imagine that his/her movement is limited by a virtual outside boundary. A simplified signal flow diagram that relates to the $C_{\alpha }$ class algorithm is illustrated in Figure 7.

#### 2.9.2. Algorithm of Class $C_{\beta }$

The class $C_{\beta }$ algorithm corresponds to major motion errors, which require a more decisive reaction. This reaction is the signal of the selected actuator (or actuators) defined by the teacher. The generation of this signal starts at a moment corresponding to a certain probe in the time pattern (the probe is selected by the teacher). An additional condition of actuator activation is the correct calculation of the time point (the decision is made on the basis of the value of the *h* distance function; see Section 2.6). The class $C_{\beta }$ algorithm is able to teach the learner to avoid typical motion errors.

## 3. Results of System Testing

### 3.1. Goals of the Experiment

The main goal of the experiment was not to assess the particular teaching algorithms, but to evaluate the general concept of algorithm selection using the results of the signal classification process. To this end, we compared two learning methods. In the first approach, the classification process was performed, and an adequate algorithm was chosen, whereas the second method used a fixed algorithm.

Learning efficiency can be assessed by the use of a previously defined benchmark, which can utilize spatial, temporal, energy, or economic aspects of the task (e.g., the level of learning of excavator control can be evaluated by the volume of extracted material over a defined time). In practice, the final benchmark is estimated by parameters and factors that correspond to certain stages of the learning process [1].

Many types of parameters, such as position matching errors, movement time, velocity, motion range, and average torques or forces [1,52], can be considered. Moreover, with digitized motion signals, we can easily calculate parameters defined in the time and frequency domain. For instance, in the time domain, the RMSE (Root Mean Squared Error) [52,53] can be calculated, whereas in the frequency domain, we can compute the mean amplitude related to certain frequency band, or the parameter IPNS (Integral of the Power spectrum density of Normalized Speed) [53]. The proper use of these parameters requires the establishment of ranges in time or space in which the parameters should be calculated. Typically, the ranges are chosen by the expert. It should be noted that the ranges can also be determined automatically using the results of the signal classification process. For instance, a signal’s belongingness to a particular class can define the time ranges in which the RMSE parameter should be computed.

In this context, the described automatic system can be regarded as a platform for evaluating the learning process. We return to these issues in Section 3.6. Here, we address the general matters of the conducted experiment.

### 3.2. Components of the Tested Learning System Prototype

**Figure 8:**
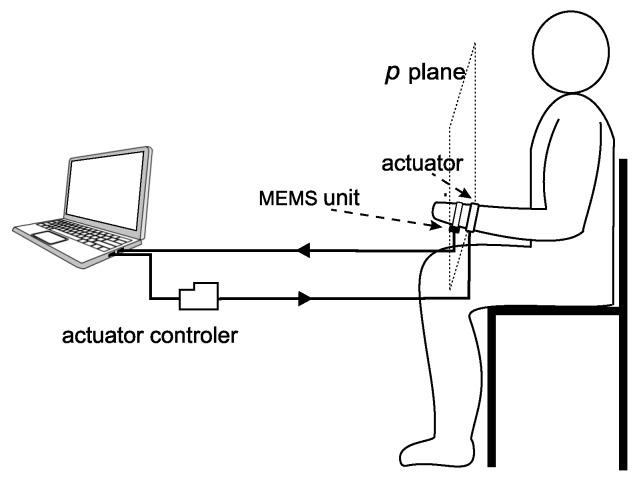
Schematic view of the system elements during the test.

In this part, we briefly depict the evaluation configuration and the system components. VN-100 [41] inertial sensors were used as motion sensors (Figure 6c). The sensors and the actuator driver were supplied with a 7.2 V battery pack. The VN-100 sensors operated at a sampling rate of 100 Hz [41], which complied with the sampling interval of the main discrete control system. The system was implemented on a portable PC minicomputer equipped with a dual core I5 processor working at a frequency of 2.4 GHz. All of the software, i.e., the signal processing and recognition modules, pattern management module, and graphical user interface, was written in C++ programming language (a small part of the software was written in the assembler). The software was developed, written, and tested by the authors (Borland C++ Builder 6.0 and Atmel Studio 7.0 environments were used). The teaching algorithms and defined signal classes are detailed in the next sections. Figure 8 presents the layout of the system elements during the experiment. The experiment was performed in a room located on the Cracow University of Technology Campus.

**Figure 9:**
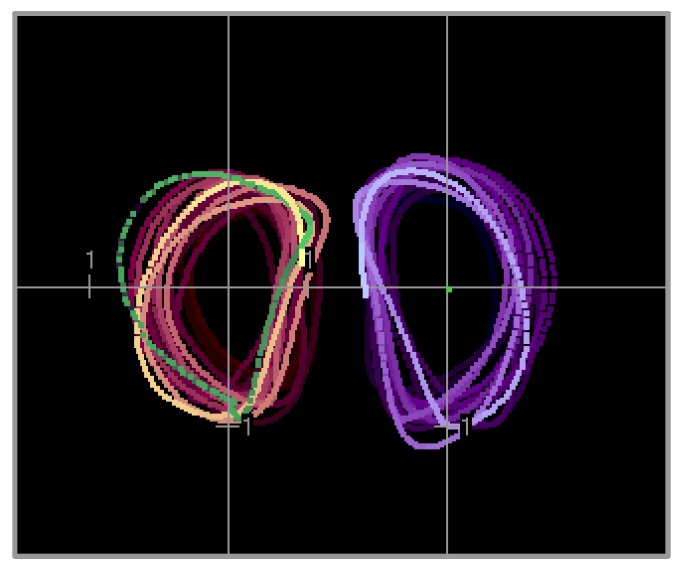
Position of the left and right wrists projected on the selected plane (it is parallel to the plane defined by the shoulder blades and tailbone). Continuously changing colors of trajectories are related to time flow; the brightest colors correspond to the latest signal probes. The units on the axes refer to 0.2 m.

From the expert’s advice, a simplified movement, which was an exercise for swimming the butterfly stroke, was chosen to be taught. The exercise consisted of moving the hand according to specific timing (with a lower speed and a pause in the upper position). Figure 9 shows the trajectories of the training motions projected on a plane that was approximately parallel to the plane through the shoulder blades and tailbone (the trajectories were displayed by a graphical user interface of the teaching system).

### 3.3. Participants

The tests were conducted using a sample of 18 students of Automatics and Robotics (Cracow University of Technology, Poland). All students participated on a voluntary basis. Before the experiment, they gave their written informed consent. The compliance of the research with ethical principles was confirmed by the institute authority responsible for research (Production Engineering Institute, Cracow University of Technology) who also supervised the research and controls publishing their results. The students (four females, 14 males) were healthy, right handed, and 22–25 years old. The exclusion criteria were as follows:

- possessing the skill of swimming using the butterfly stroke,
- lack of normal motor control of the upper extremities,
- a history of neurological disorders (declared by the subject).

The participants were randomly divided into two groups of the same cardinality.

### 3.4. Teaching Algorithms and Signal Classes Used in the Test

The pattern of the correct motion ($C_{\alpha }$ class) was created by the expert (swimming instructor), whereas the pattern of the $C_{\beta }$ class was created by an additional student selected by the expert using a short preliminary test. The selected person demonstrated typical errors consisting of the lack of the desired pause in the upper phase of the movement (the person did not participate in further research). The $C_{\beta }$ class algorithm generated actuator impulses at the moment at which the pause should occur.

### 3.5. Test Procedure

The total test duration was limited to five minutes (plus a break) to prevent the subjects from tiredness and to test only the transfer of skills [1]. For both groups of participants, the course of learning was as follows:

First phase (lasting 1 min): use of the automatic teaching system, which was supervised

by the instructor,

Second phase (2 min): normal operation of the system (without instructor),

Third phase (3 min): break,

Fourth phase (2 min): examination of the learned skills, unassisted repetition of the exercise

by the learner (actuators were switched off).

The first group was taught using the method that applied the classification process to select the proper algorithm (related to $C_{\alpha }$ or $C_{\beta }$), whereas the second group was taught using the class $C_{\alpha }$ algorithm only (without the classification process).

### 3.6. Outcome Measures and Data Analysis

To evaluate learning quality, we used three different parameters. The first factor was based on the RMSE parameter [53] and estimated the accuracy of the motion learned by the person [1,52]. We calculated the RMSE from each preprocessed one-dimensional signal $S_{i}=\left(s_{i}^{1},s_{i}^{2},\ldots ,s_{i}^{m}\right)$ (2), which corresponded to a position. Each parameter was computed in relation to the relevant shape pattern: $P_{i}=\left(p_{i}^{1},p_{i}^{2},\ldots ,p_{i}^{w}\right)$ (3) (in our case, two shape patterns for the *x* and *z* positions were defined). The $i^{\mathrm{th}}$ RMSE parameter was computed by:

(15) $${\mathrm{RMSE}}_{i}={\left(1/n\sum_{k=1}^{n}{\left(s_{i}^{k}-p_{i}^{ind(k)}\right)}^{2}\right)}^{1/2}$$

where *n* is the length of the range in which RMSE${}_{i}$ is calculated and ind(k) is the index of the pattern probe that corresponds to the $k^{\mathrm{th}}$ signal probe.

The function ind(k) computes the index of the shape pattern using the method of calculating the time point (Section 2.6). From all the calculated RMSEs, the arithmetic mean was computed. This value, denoted by $E_{1}$, estimated the aggregate deviation of the movement. It was computed in the defined range of the signal (from 0.3 to 0.9 of its total length), which referred to the fourth phase of the experiment.

The second parameter, denoted by $E_{2}$, was similarly computed; however, the signal classification process was used to establish a suitable time range. First, the longest time interval in which the motion signal belonged to only the $C_{\alpha }$ class was determined. The beginning of this interval was the starting point of the range in which the parameter was calculated (the range had a constant length of 30 s). Because the best range was determined, this factor was, to some degree, resistant to accidental errors of motion.

The last parameter, denoted by $E_{3}$, was used to evaluate the progress of learning. This can be expressed by a difference between the values of selected parameters that correspond to the testing phase and a certain stage before learning (or its early phase) [52]. The simple difference can be extended by a weight factor that determines the impact of the earlier phase. In our case, the $E_{3}$ parameter was computed as follows:

(16) $$E_{3}=E_{2}^{test}-\beta E_{2}^{pre}$$

where $E_{2}^{test}$ and $E_{2}^{pre}$ are parameter values corresponding to the fourth (test) and first phases of the experiment, respectively, and β=0.25.

The calculated values of the parameters $E_{1}$, $E_{2}$, and $E_{3}$ for participants of both groups are sequentially presented in Table 3.

The obtained results can be analyzed using Student’s *t*-test (two-sample location test). However, we should ensure that suitable conditions of *t*-test usage are met. The groups of data, corresponding to two learning methods, should be sampled independently, have a normal distribution, and have equal (homogeneous) variances. In our case, we checked whether the data had a normal distribution (normality test) using the Shapiro–Wilk test. Next, the homogeneity of the variance was assessed using Levene’s test.

The calculated values of statistics referred to Shapiro–Wilk and Levene’s tests, as well as their critical values related to a confidence level of 0.95 are shown in Table 4.

On the grounds of the obtained results, we applied Student’s *t*-test to all efficiency parameters. Table 4 presents the mean values of $E_{1}$, $E_{2}$, and $E_{3}$; their standard deviations; the calculated values of Student’s t-distribution; and the *p*-values that correspond to them.

### 3.7. Test Results and Discussion

Based on the results of Student’s *t*-test, with a probability of error of 0.049, 0.046, and 0.042 for the efficiency parameters $E_{1}$, $E_{2}$, and $E_{3}$, respectively, the hypothesis stating that the mean efficiency of both learning methods is equal should be rejected in favor of the hypothesis that the learning efficiency is greater when the classification process is used. However, this positive result should be discussed in light of several aspects.

#### 3.7.1. The High Level of Motion Errors

Let us focus on the $C_{\alpha }$ class algorithm. The vector of the error e was calculated at exact moments (Section 2.7). Thus, a significant error value may appear when a movement is performed too early or too late, even if the motion is correct. Consequently, the class $C_{\alpha }$ algorithm sends corrective signals that refer to not only spatial, but also temporal errors of the movement. This effect was observed in the upper area of the learned movement in which the short pause should occur. Most often, the output signals were generated just before or after the expected moment of pause.

Additionally, taking into account a variable delay of human reactions, the whole system can become unstable, as defined in control system theory [12]. As a consequence, we obtained a relatively high level of motion errors for all the parameters ($E_{1}$ = 94.5, $E_{2}$ = 95.8, and $E_{3}$ = 70.3). This can be considered a significant drawback of the $C_{\alpha }$ class algorithm.

These errors can be corrected by applying an additional control algorithm that can interrupt the process of generating improper and confusing output signals. In fact, we observed that applying the $C_{\beta }$ class algorithm (in addition to the $C_{\alpha }$ algorithm) led to a reduction in the mean errors (for the method that uses both the algorithms, the efficiency parameters were $E_{1}$ = 71.1, $E_{2}$ = 69.1, and $E_{3}$ = 40.0). Improving of all the parameters (particularly the $E_{3}$ that assesses the learning progress) can be regarded as a substantial advantage of the proposed method.

#### 3.7.2. The Problem of Properly Interpreting Haptic Messages

The observation of the tests revealed that many subjects had significant problems with the proper interpretation of fast messages from vibrotactile actuators (it is “hard to follow”, they reported). The calculated values of Student’s t distributions (1.75, 1.8, 1.84, for parameters $E_{1}$, $E_{2}$, and $E_{3}$) were close to the critical value of 1.746, related to confidence level of 0.95. We can expect that this level will be exceeded for faster moves. Therefore, to teach very fast movements, specialized teaching algorithms must be developed. Moreover, some subjects exhibited exceptionally low sensitivity to the haptic stimulus (e.g., Participant No. 2 from the first group and No. 4 from the second). For these persons, as above, specialized algorithms for teaching should be created. We should also note that generating messages too frequently had a surprisingly large effect on the proper detection of the messages (the time between the actuators’ activation should exceed 2 s, a relatively high value).

The communication between the system and the learner may be supported by the use of other senses (hearing, sight). However, because we aimed to create a system for teaching fast motions, such as those in sports and machine operation, this solution was intentionally disregarded. Such applications require learners to focus their visual and mental attention on the particular task performed.

#### 3.7.3. Creating Efficient Teaching Systems

The possibility of creating an effective automatic system that uses vibrotactile feedback and is applicable to sports has been discussed in the literature. The current status of the research was concisely articulated in the quotation from [14]: “Haptic feedback: Many concepts, few proofs”. In the above discussion, we argue that the efficiency of teaching fast movements can be improved by using the classification process to determine the learning algorithm. This can be regarded as the main outcome of the presented research.

This result can help to develop miniaturized “personal” teaching systems for sports and rehabilitation applications. In this case, the classification task was relatively simple and can be performed using HMM methods. The low time consumption of these methods (Table 2) enabled their implementation without the use of high performance processors. However, because of the difficult supervision of the classification phase, developing larger systems may be impossible. The presented research showed that the kNNModel method enabled creating sophisticated teaching systems that were equipped with several sensors and actuators. Such systems can be embedded in the steering systems of machines (e.g., excavator or crane) and vehicles. In this way, machine operators can be trained in a near-real working environment.

#### 3.7.4. Expert’s Knowledge and Problems of System Optimization

The knowledge included in the motion patterns is an essential part of the teaching system. However, we must emphasize that this knowledge comes from an expert, which can be considered an external element. This inhomogeneous system structure is difficult to model and precludes using conventional methods of synthesis and optimization of the control systems, such as the Markov Decision Process (DDP) or the Iterative Linear Quadratic Regulator (ILQR) [12].

## 4. Conclusions

The creation of an automatic system for teaching motion activities, especially related to sports or professional work, is a difficult problem. The main goal of this study was to show that the proposed automatic system was able to carry out the teaching process effectively. The conducted tests confirmed this thesis. The most essential features of the described system were the application of MEMS inertial sensors in the process of motion capture, the use of vibrotactile actuators to ensure system–learner communication, and the application of the motion classification process to select the adequate teaching algorithm.

Additionally, this paper described several solutions that aided in the creation of an efficient system, particularly the teacher’s choice of the appropriate system configuration (including the kind of motion signals used), calculating actuator signals as linear combinations of the desired motion components, and the use of the results of the signal classification to calculate the learning efficiency parameters. The articulation of the above issues (especially from the first group) was an attempt to formulate a standardized approach to building a motor learning system.

The results of the performed test showed that subjects had significant difficulties in correctly interpreting fast haptic messages. A solution to this problem, as proposed in this paper, is building specialized algorithms for teaching fast motion activities. This defines the basic scope of the future works. Additional system improvements should be the development of advanced methods for creating and editing motion patterns (e.g., automatically matching the patterns to individuals) and the addition of a module to manage a database with patterns of typical motion activities. 

## Figures and Tables

**Table 1:** A brief review of selected teaching systems using different paradigms and approaches, including sensor types, methods of communication with the user, and the aim and scope of the analysis.

Authors and Works	Application Field	Sensors	Communications to Learners	Scope of Analysis
Zahradka, Behboodi et al. [19], 2019	neuromuscular rehabilitation	MEMS IMU	functional electrical stimulation	online gait phase detection
Bark, Hyman [18], 2015	rehabilitation after stroke	infrared camera	haptic, visual	position controlling
Haladjian, Reif, BrĂĽgge [20], 2017	rehabilitation, sports skiing	MEMS IMU	haptic	guiding for visually impaired skiers
Taborri, Palermo, Rossi et al. [21], 2019	sports race walking	MEMS IMU	without communication	offline classification for referee’s and trainer’s analysis
Alonso, Dieguez et al. [16], 2015	sports volleyball	biometric sensors	without communication	online classification for trainer’s analysis
Stamm [6], 2018	sports swimming	MEMS IMU	without communication	offline analysis for trainer
Wang, Wang, Zhao et al. [22], 2019	sports swimming	MEMS IMU	without communication	offline analysis of movement parameters
Umek, Kos et al. [7], 2018	sports swimming, kayaking	MEMS IMU	without communication	online monitoring for trainer’s analysis
Jiao, Wu, Bie, Umek, Kos [23], 2018	sports golf	MEMS IMU	without communication	offline classification for trainer’s analysis
Moeyersons, Fuss, Tan, Weizman [5], 2016	sports snowboarding	pressure sensors	haptic, visual	trainer’s online analysis and feedback
Hachaj, Ogiela, Piekarczyk [24], 2014	sports karate	multiple infrared depth cameras	without communication	online classification for trainer’s analysis
Hachaj, Piekarczyk, Ogiela [25], 2017	sports karate	MEMS IMU	without communication	offline classification for trainer’s analysis
Wang, Yao et al. [9], 2017	surgical training	MEMS IMU joystick	haptic, visual	online surgery simulation and analysis
Żywicki, Zawadzki, Górski [8], 2017	work skills training (Industry 4.0)	MEMS IMU	haptic, visual	online simulation of operation in factory

**Table 2:** Results of the numerical experiments and the main properties of the HMM and minimal distance kNNModel methods. The classification was performed on nine minute signal divided into two parts referring to two classes of signals (Section 2.9). In order to apply the HMM method, these parts are segmented into two second fragments corresponding to signal periods. In the HMM approach, Nis the number of states and T is the length of the string of symbols describing the signal fragments; in the kNNModel, T is the number of probes in the compared fragments of the signal. The features of the kNNModel method strongly depend on the defined distance function (see (9)).

Method	Time Complexity of the Classifier	Classification Time (ms)	Classification Error Level (%)	Possibility to Interpret
HMM	$O(N^{2}T)$	0.003	14	possible, but difficult
kNNModel	$O(T^{2})$	3.7	11	easy

**Table 3:** $E_{1}$, $E_{2}$, and $E_{3}$ are parameters that evaluate teaching efficiency for the two methods of learning (method =1 includes the classification process); the unit of all parameters is 1 mm.

Parameter	Method	Participant Index in the Group								
		1	2	3	4	5	6	7	8	9
$E_{1}$	1	63	102	80	79	57	73	57	75	56
	2	137	87	102	160	72	77	69	96	51
$E_{2}$	1	50	109	80	81	41	79	63	67	52
	2	129	99	131	156	54	79	65	96	54
$E_{3}$	1	16	92	44	59	11	64	45	45	22
	2	104	74	93	122	41	44	40	74	40

**Table 4:** Results of the Shapiro–Wilk (S-W) test, Levene’s test, and Student’s *t*-test for the parameters $E_{1}$, $E_{2}$, and $E_{3}$ and the two learning methods.

Parameter	Method	Mean (mm)	Std. dev. (mm)	S-W	S-W crit.val.	Levene	Levene crit. val.	Student’s t Distribution	Student’s t *p*-Value
$E_{1}$	1	71.1	15	0.89	0.83	3.5	4.5	1.75	0.049
	2	94.5	35	0.92					
$E_{2}$	1	69.1	21	0.95		3.2		1.80	0.046
	2	95.8	37	0.93					
$E_{3}$	1	44.0	26	0.94		0.9		1.84	0.042
	2	70.3	31	0.87					
